# Determination of the frequency of individuals with broadly cross-reactive neutralizing antibodies against PRRSV in the sow population under field conditions

**DOI:** 10.1186/s40813-024-00372-y

**Published:** 2024-07-08

**Authors:** Ángeles Plaza-Soriano, Francisco Javier Martínez-Lobo, Laura Garza-Moreno, Jaime Castillo-Pérez, Elki Caballero, José María Castro, Isabel Simarro, Cinta Prieto

**Affiliations:** 1 SALUVET group, Animal Health Department, Faculty of Veterinary Medicine, Universidad Complutense de Madrid, Madrid, Spain; 2 Animal Science Department, School of Agrifood and Forestry Engineering and Veterinary Medicine, University of Lleida, Lleida, Spain

**Keywords:** Porcine reproductive and respiratory syndrome virus, Broadly neutralizing antibodies, Elite neutralizers

## Abstract

**Background:**

Porcine Reproductive and Respiratory Syndrome Virus (PRRSV) is a significant swine pathogen, yet the immune response components contributing to protection remain incompletely understood. Broadly reactive neutralizing antibodies (bNAs) may play a crucial role in preventing reinfections by heterologous viruses, although their occurrence is considered low under both field and experimental conditions. This study aimed to assess the frequency of sows exhibiting bNAs against PRRSV under field conditions and to analyze the epidemiological factors influencing the occurrence of these elite neutralizers. Blood samples were collected from breeding sows across eleven unrelated pig farms, with samples categorized by parity. Serum obtained was utilized in virus neutralization assays (VNs) against six PRRSV field isolates and two MLV strains.

**Results:**

Approximately 7% of the sows exhibited neutralization activity against all viruses in the panel, with a geometric mean of the titer (GMT) of NAs at or exceeding 4 log_2_. Exclusion of the PRRSV-2 isolate from the panel increased the proportion of elite neutralizers to around 15%. Farm-specific analysis revealed significant variations in both GMT of NAs and proportion of elite neutralizers. PRRSV unstable farms and those with a PRRS outbreak in the last 12 months displayed higher GMT of NAs compared to stable farms without recent outbreaks. The GMT of NAs showed a gradual, albeit moderate, increase with the parity of the sows. Parity’s impact on bNA response was consistently observed in stable farms but not necessarily in unstable farms or those with recent outbreaks. Finally, the results indicated that vaccinated animals had higher NA titers against the vaccine virus used in the farm than against field viruses.

**Conclusion:**

bNAs against heterologous isolates induced by PRRSV infection under field conditions are generally low, often falling below titers necessary for protection against reproductive failure. However, a subset of sows (approximately 15%) can be considered elite neutralizers, efficiently recognizing various PRRSV strains. Repeated exposures to PRRSV play a crucial role in eliciting these bNAs, with a higher frequency observed in unstable farms and those with recent outbreaks. In stable farms, parity only marginally influences bNA titers, highlighting its limited role compared to the impact of PRRSV exposure history.

**Supplementary Information:**

The online version contains supplementary material available at 10.1186/s40813-024-00372-y.

## Background

Porcine Reproductive and Respiratory Syndrome (PRRS) is currently recognized as one of the most significant endemic diseases of swine, probably because of its huge economic impact in the pig industry [1, 2]. PRRS only affects suids and the infection causes reproductive failure in pregnant females, characterized by abortions, premature farrowings and full-term litters composed by stillborns, weak-born piglets and mummified fetuses [3], and respiratory disorders in growing pigs [4].

The condition is caused by PRRS virus (PRRSV), an RNA virus classified in the newly created genus *Rodartevirus* of the family *Arteriviridae*, within the order *Nidovirales* [5]. One of the most outstanding characteristics of PRRSV is its genetic variability that has prone the division of PRRSV strains into two different species: PRRSV-1 (i.e. the former genotype 1 or European type) and PRRSV-2 (i.e.. the former genotype 2 or American type) [6]. Likewise, big differences in the nucleotide sequence have been described within each species [7–9]. This genetic variability is considered to be responsible for the significant antigenic differences documented not only between PRRSV-1 and PRRSV-2 but also between isolates of the same PRRSV species [10–12].

Furthermore, PRRSV variability, and more specifically antigenic variability, has hindered the development of effective control strategies against the disease, and protection achieved by vaccination, or even previous exposure to the virus, is often partial [13]. This is most likely due to the lack of recognition of antigenic determinants of heterologous viruses. Thus, although passive transfer studies have demonstrated that neutralizing antibodies (NAs) might play a key role in protection against re-infections [14], NAs developed upon infection are mainly strain-specific, with very limited capacity of heterologous recognition [15]. Yet, some sera exhibit an outstanding neutralizing activity against heterologous strains, which seems to indicate that the NAs developed by those individuals are directed against conserved epitopes [15, 16]. This phenomenon has been described for other viral pathogens as Human Immunodeficiency Virus type 1 (HIV-1), Influenza Virus (IV) or Hepatitis C Virus (HCV) [17–20]. Those broadly reactive NAs (bNAs) might play a role in protection and in the avoidance of reinfections by heterologous viruses in the case of highly variable viruses [19, 20]. Similarly, in the case of PRRSV, the presence of significant amounts of bNAs seems to be effective in protection, even against heterologous challenges [21, 22].

However, bNAs are infrequent upon infection. In the case of HIV-1 the proportion of individuals that develop broad and very potent NAs that can neutralize a wide range of genetically diverse HIV-1 subtypes is only about 1% [17]. Similarly, only 0.6% individuals had broad neutralizing activity against diverse Human Cytomegalovirus (HCMV) strains [23] and 2-5% of HCV-infected individuals demonstrated outstanding HCV-neutralizing activity [24]. Recently, exceptional neutralization activity against severe acute respiratory syndrome coronavirus 2 (SARS-CoV-2) has been described in 3% of infected individuals [25].

Likewise, the proportion of PRRSV-2 infected animals that exhibit bNAs is considered low under field conditions [15]. In the same way, experimental studies indicate that only roughly 1% of the pigs vaccinated and challenged develop broadly NAs able to recognize efficiently six genetically diverse viruses in virus neutralization (VN) assays [26]. However, not much information is available in relation to PRRSV-1 and this information derives from experimental studies [15].

Several factors might condition the ability of an individual to develop these bNAs, including host characteristics, as genetic background and haplotype, virus characteristics (i.e. individual properties of the strain causing the infection or used for immunization) and frequency of exposure to the virus [16, 27]. Thus, the determination of the factors affecting the development of bNAs might be relevant for the stimulation of a better protection in the population. Consequently, the main objectives of this study were to determine the frequency of sows exhibiting bNAs against PRRSV-1 under field conditions and to characterize the epidemiological factors that can have an influence on the frequency of the so-called elite neutralizers, i.e. animals that develop bNAs that can neutralize most PRRSV isolates.

## Methods

### Selection of the farms and sampling

Eleven PRRSV positive sow farms were selected based on the genetics of the population, the vaccination protocol implemented in each farm, the PRRSV stability status and their PRRS outbreak history (Table 1).

**Table 1:** Characteristics of pig farms selected for the study

Farm	Genetic Line	Vaccination	PRRS status at the time of sampling	PRRSV outbreaks during 12 months before sampling
A	Iberian	None	Positive stable	No outbreak
B	UPB Genetic World	Vac-1^a^	Positive unstable	Outbreak
C	UPB Genetic World	Vac-3^b^	Positive unstable	No outbreak
D	TOPIGS NORSVIN	Vac-3	Positive stable	No outbreak
E	TOPIGS NORSVIN	Vac-3	Postive stable	No outbreak
F	PIC	Vac-1	Positive stable	No outbreak
G	JSR Genetics	Vac-3	Positive stable	Outbreak
H	DanBred	Vac-1	Positive stable	Outbreak
I	DanBred	Vac-1	Positive unstable	Outbreak
J	PIC	Vac-1	Positive unstable	Outbreak
K	Iberian	None	Positive stable	No outbreak

^a^: MLV, Unistrain®PRRS, HIPRA Laboratories; ^b^: MLV, Porcilis® PRRS, MSD Animal Health

Thus, the experimental design was set to include a representation of the sow genetics most frequently used in Spain as Danbred, PIC, Topigs, UPB, JSR and Iberian pigs. In most of those farms, a PRRSV-1 MLV blanket vaccination every three or four month protocol of adult sows was implemented. Besides, in vaccinating farms, all the gilts were purchased from PRRSV-negative sources and vaccination with MLV was used for acclimatization period. On the contrary, two farms were PRRSV positive but did not vaccinate incoming gilts or adult sows.

Farms were classified as stable or unstable and as “outbreak” or “no-outbreak” by their private veterinary services. Stability was assessed following the criteria established by Holtkamp et al. [28]. Thus, 30 serum samples were taken weekly from due to wean piglets and tested against PRRSV in pools of 5 by RT-qPCR. A farm was classified as stable when PRRSV was not detected in any of the samples taken in the previous 90 days. On the contrary, a farm was classified as unstable when PRRSV was detected in any serum sample obtained from due to wean piglets in the same period. On the other hand, farms were classified as “outbreak” when clinical signs suggestive of PRRSV infections, including increased abortions, off-feed sows, stillbirths, mummies, and preweaning mortality, were observed in the breeding herd in the previous 12 months and as “no-outbreak” when no clinical signs of PRRSV infection had occurred in the same period of time. Occasionally, a farm had experienced a PRRSV outbreak in the last 12 months but had already reached stability by the time the farm was sampled for this study. In that case the farm was defined as stable and “outbreak”.

The expected proportion of elite neutralizers (roughly 10%) was used to calculate the sample size needed in this study. This figure was based on the results of previous studies carried out by the research group using experimentally immunized pigs [15]. Then, considering a binomial distribution and a population size between 1000 and 3000 sows (the herd size on sampled farms ranged from 1200 to 2800 sows), a sample size of 60 sows targeting different parities was selected (confidence interval equal to two margins of errors; 95% confidence). Thus, 10 sows of 1st, 2nd, 3rd, 4th, 5th and 6th or more parities were randomly selected and sampled per farm, with the exception of Farm A in which 57 sows were sampled.

### PRRSV isolates and cell cultures

Eight PRRSV isolates were used in the VN assays. Five were PRRSV-1 field strains, one was a PRRSV-2 field strain, which was used to determine the ability of the sera to recognize highly heterologous viruses, and the remaining two viruses were vaccine strains included in two MLV PRRSV-1 commercial vaccines. These two vaccine strains were included in the study because they were used for the immunization of incoming gilts and sows in the selected farms and are widely used in Spain. The field virus strains were selected based on their susceptibility to neutralization against a panel of monospecific hyperimmune sera [15]. The objective was to include viruses that differ in their susceptibility to neutralization to better represent the myriad of viruses circulating in the pig population (Table 2).

All PRRSV isolates were cultured and titrated as previously described by Scortti et al., 2006 [29] and Reed and Muench method [30].

**Table 2:** PRRSV isolates used in the study

Isolate	Specie/Subtype	Country	Year of isolation	Sensitivity to neutralization (Tier)^1^	ORF5 GenBank accession numbers^3^
Sp-2a	PRRSV-1/Subtype1	Spain	1991	Sensitive (2 A)	JF730961
Sp-3a	PRRSV-1/Subtype1	Spain	1992	Resistant (4)	JF730962
EU-7a	PRRSV-1/Subtype1	Belgium	1996	Moderately resistant (3)	JF730990
EU-11a	PRRSV-1/Subtype1	Czech Republic	1996	Very sensitive (1)	JF730993
EU-18a	PRRSV-1/Subtype1	Italy	2002	Resistant (4)	JF730999
AM-5a	PRRSV-2	USA	1996	Resistant (4)	AY545985
Vac-1	PRRSV-1/Subtype1	Spain	1992	NA^2^	MK134483
Vac-3	PRRSV-1/Subtype1	The Netherlands	1992	NA^2^	AY743931

^1^: As described by Martínez-Lobo et al., (2011) [15] ^2^: Not available ^3^: ORF5 identity matrix is depicted in Additional File 1

### Viral neutralization assays

To determine the presence of PRRSV-specific NAs in the collected sera the abovementioned viruses were used in VN assays following a technique previously described [15]. Briefly, serum samples were inactivated at 56 °C for 30 min and serially diluted two-fold with fresh cell-culture medium without FBS in 96-well tissue culture plates. Then, a viral suspension containing 100 TCID_50_ of the appropriate PRRSV isolate was added to each well and the mixture incubated for 1 h at 37 °C. Thereafter, a suspension of MARC-145 cells was added to each well and the plates were incubated at 37 °C in humidified atmosphere containing 5% CO_2_ for six days. All samples were analyzed in duplicate. The culture plates were examined for cytopathic effect on days 4, 5 and 6 post-inoculation. All samples were analyzed in duplicate and the same viral batch was used to test all sera. The titers of NAs were expressed as log_2_ of the reciprocal of the serum dilution that completely inhibited viral replication in 50% of the wells.

### Analysis of the sera characteristics

The results of the VN assays were used to determine the breadth and the potency of each serum, parameters that have been used to characterize neutralization responses across the virus diversity, especially in the case of HIV [31], and more recently SARS-CoV-2 [32]. The breadth indicates the ability of each specific serum to react with different viral isolates. Thus, this parameter is defined as the percentage of isolates that are neutralized by each serum, regardless the serum dilution at which the virus is neutralized.

The potency is defined as the mean titer at which each serum neutralizes the virus isolates of the panel. Consequently, this parameter indicates the amount of NAs with heterologous neutralization capacity present in each serum. To determine the potency, the geometric mean of the titer (GMT) at which each serum neutralized the set of viral isolates was calculated, assigning an arbitrary value of 0.1 to the sera without detectable neutralizing activity against a particular virus.

A sow was defined as elite neutralizer when its serum recognized at least all PRRSV-1 isolates of the panel and the GMT of NA was ≥ 4 log_2_.

### Statistical analysis

Differences in the GMT of NA were evaluated for significance using Kruskal–Wallis’ non-parametric and Dunn’s multiple comparisons tests. The assessment of differences in the proportion of elite neutralizers for each factor studied was carried out using the Chi square (χ^2^) test and the Fischer F test.

In both cases, a *p* value < 0.05 was considered statistically significant.

## Results

### PRRSV NA titers and proportion of elite neutralizers in the population

**Fig. 1:**
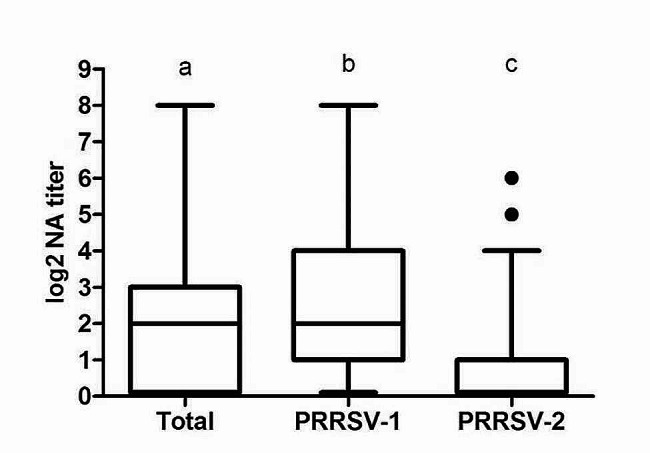
GMT of NAs in the sera of the sampled sows against the PRRSV isolates used in this study. Each box represents the range between 25 and 75% of the observations. The line inside each box represents the median. The whiskers above and below each box extend up to 1.5 times the interquartile range (ICR). Points represent outliers. Different letters on each caterogy indicate statistically significant differences

The GMT of NAs in the population studied is represented in Fig. 1. NA titers were, in general, very low. Thus, the GMT of NAs against all the viruses used in this study (global) was 2.12 log_2_, while a slightly higher GMT was obtained when only PRRSV-1 were considered in the analysis (2.43 log_2_). In contrast, the response against the only PRRSV-2 virus included in the panel was very poor. Most sera did not recognize this virus and the GMT of NAs against the PRRSV-2 isolate was 0.71 log_2_. However, it is remarkable that a small proportion of individuals, i.e. 17.35%, were able to recognize and neutralize efficiently this PRRSV-2 isolate. The differences in the GMT of NAs against global, PRRSV-1 and PRRSV-2 viruses were statistically significant (*p* < 0.05), which indicates that PRRSV-1 isolates were better recognized by the sera used in this study.

The distribution of the sows based on the GMT of NAs obtained is summarized in Table 3. As it can be observed in the table, the GMT of NAs varied significantly between sows, although most animals exhibited modest mean values. Specifically, roughly one third of the sows had GMT of NAs ≤1.0 log_2_ and up to 71.23% ≤3.0 log_2_ (equivalent to 1:8). Nevertheless, some individuals presented higher values. Thus, the GMT of NAs of 15.98% of the sows exceeded 4 log_2_ and they were considered elite neutralizers and up to 3% exceeded the titer of 6 log_2_.

**Table 3:** Distribution of sows (number and percentage of the total population they represent) based on their GMT of NA against all PRRS viruses in the panel

NA titer (log_2_)	Number of sows	Percentage
0.1-1.0	218	33.18
1.1-2.0	128	19.48
2.1-3.0	122	18.57
3.1-4.0	84	12.79
4.1-5.0	52	7.92
5.1-6.0	33	5.02
6.1-7.0	11	1.67
7.1-8.0	9	1.37
**TOTAL**	**657**	**100**

The NA response against the different PRRSV-1 isolates differed significantly (Additional file 2). Isolates EU-9a and EU-11a exhibited the highest sensitivity to neutralization, with GMT of NA slightly above 3 log_2_. Conversely, GMT of NAs against isolates Sp-3a and EU-18a were notably low. These results are consistent with the susceptibility to neutralization of the PRRSV-1 isolates selected for this study [14].

### Determination of the influence of the farm characteristics on the PRRSV NA titers and in the proportion of elite neutralizers

**Fig. 2:**
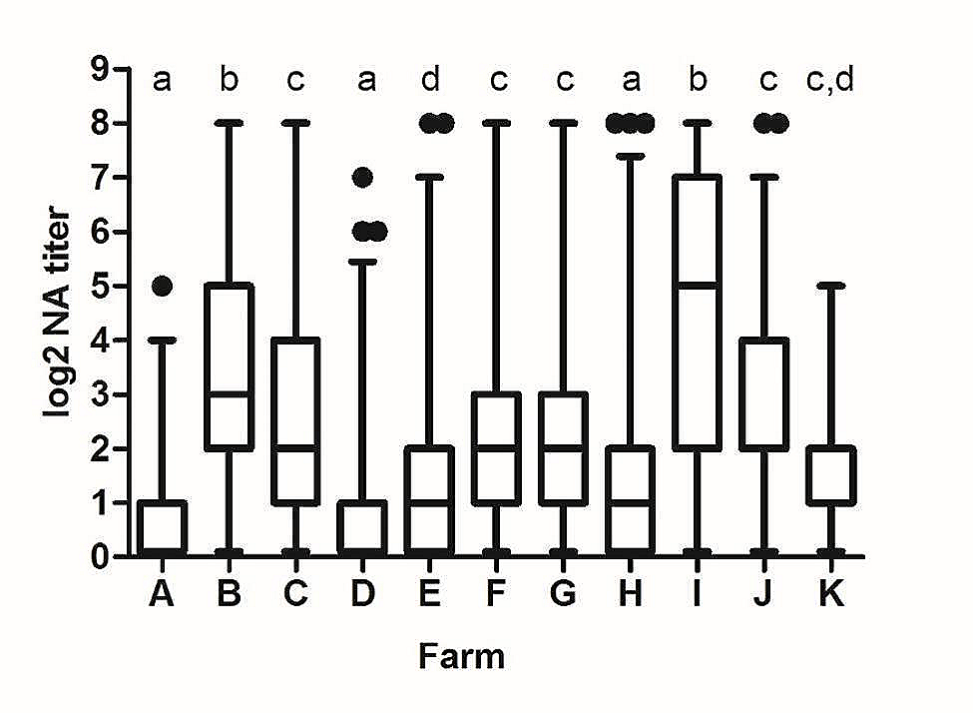
GMT of NAs against the viral isolates used in the study per farm. Each box represents the range between 25 and 75% of the observations. The line inside each box represents the median. The whiskers above and below each box extend up to 1.5 times the interquartile range (ICR). Points represent outliers. Different letters on each farm indicate statistically significant differences

When the GMT of NAs obtained in the different farms were compared, statistically significant differences were observed (*p* < 0.05) (Fig. 2). As it can be observed in the figure, the GMT of NAs was very low in farms A, D, E and H, with values around 1.0 log_2_, which is the cut-off point of the VN assay. Farms C, F, G, J and K exhibited GMT of NAs values slightly higher, but still low, in a range from 1.0 to 2.0 log_2_. On the contrary, two farms, farm B and farm I, presented GMT of NAs values greater than 3.0 log_2_. These differences were particularly notable between farms A and D, which had exceptionally low values, and farms B and I, in which the GMT of NAs was substantially higher than in any other herd.

**Fig. 3:**
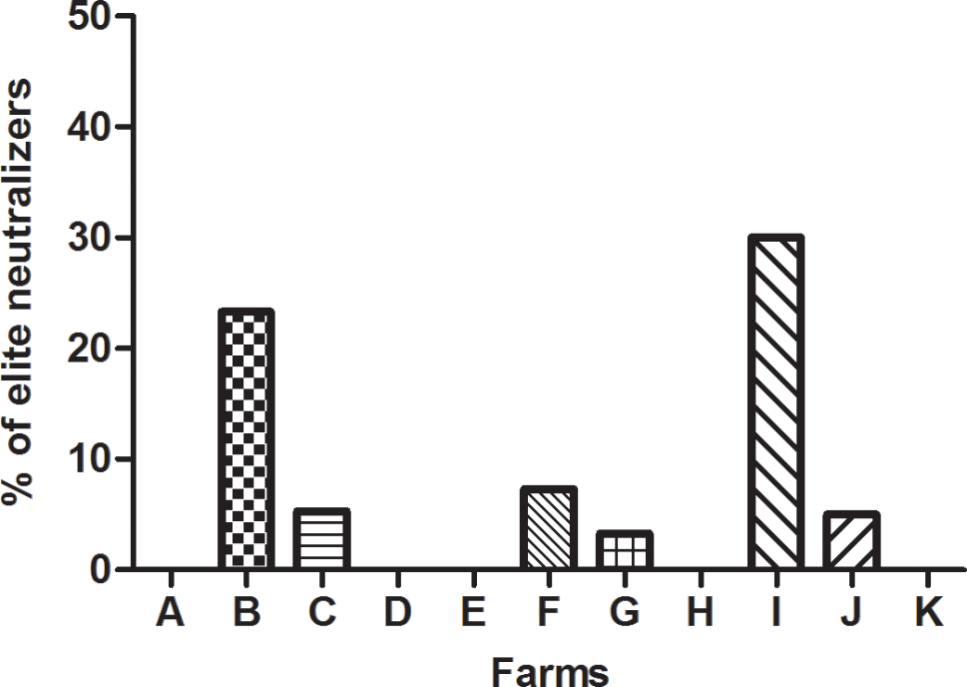
Percentage of sows considered elite neutralizers per farm

The higher GMT of NAs found in farms B and I corresponded with a high proportion of elite neutralizers in those farms (23.3 and 30.0%, respectively), while low GMT of NAs generally led to an absence of elite neutralizers on those farms (Fig. 3). Thus, no elite neutralizers were identified in farms A, D, E and K. The marked differences in the proportion of elite neutralizers between farms were statistically significant when farms B and I were compared to the rest of the farms (*p* < 0.05).

**Fig. 4:**
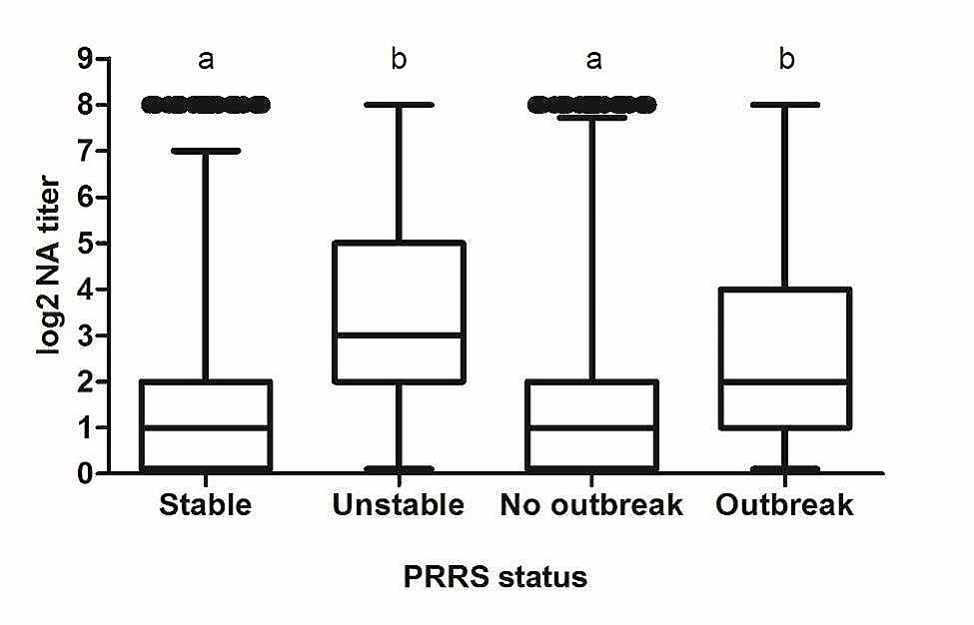
GMT of NAs against the viral isolates used in the study according to the farm PRRSV status. Each box represents the range between 25 and 75% of the observations. The line inside each box represents the median. The whiskers above and below each box extend up to 1.5 times the interquartile range (ICR). Points represent outliers. Different letters on each status indicate statistically significant differences

In addition, the influence of the PRRSV status (i.e. PRRSV stability and the occurrence of PRRS outbreaks in the last 12 months) on the GMT of NAs values was studied. The results obtained indicated that farms in which the virus was actively circulating or where recent PRRS outbreaks had occurred, the GMT of NAs were higher than in stable or no-outbreak farms (Fig. 4). The differences were statistically significant (*p* < 0.001). On the contrary, no differences were observed between breeds in the GMT of NAs values or in the percentage of elite neutralizers.

**Fig. 5:**
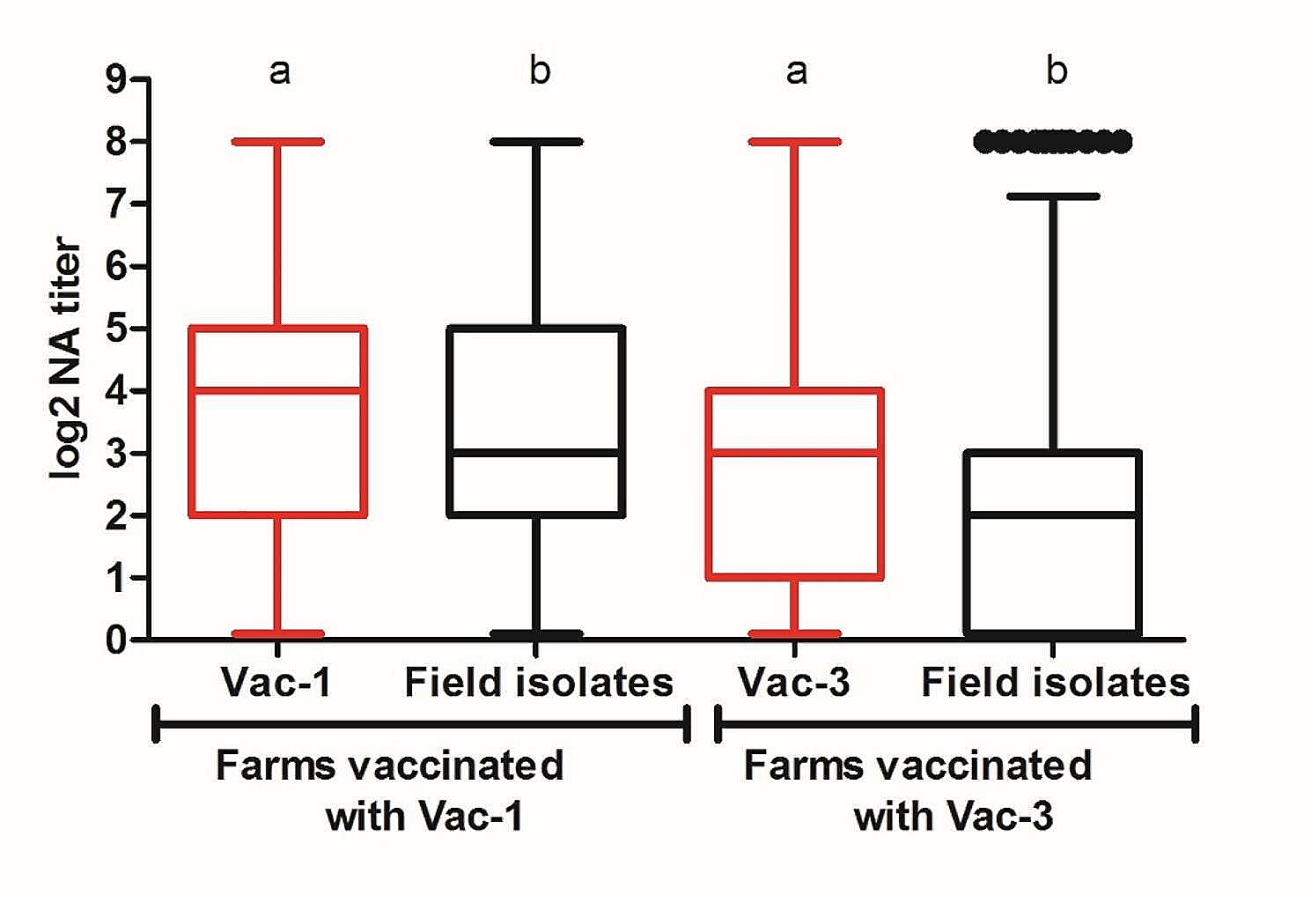
GMT of NAs against the vaccine used on the farm and against the PRRSV-1 field isolates used in the study. Each box represents the range between 25 and 75% of the observations. The line inside each box represents the median. The whiskers above and below each box extend up to 1.5 times the interquartile range (ICR). Points represent outliers. Different letters indicate statistically significant differences

Finally, in the vaccinated farms, the GMT of NAs against the vaccine strain used in that farm was compared to the GMT of NAs obtained against all PRRSV-1 field viruses used in the study. The results showed that the response of NAs was systematically higher against the vaccine strain than against the field viruses.(Fig. 5.).

### Determination of the influence of the sow parity on the titer of NAs against PRRSV

**Fig. 6:**
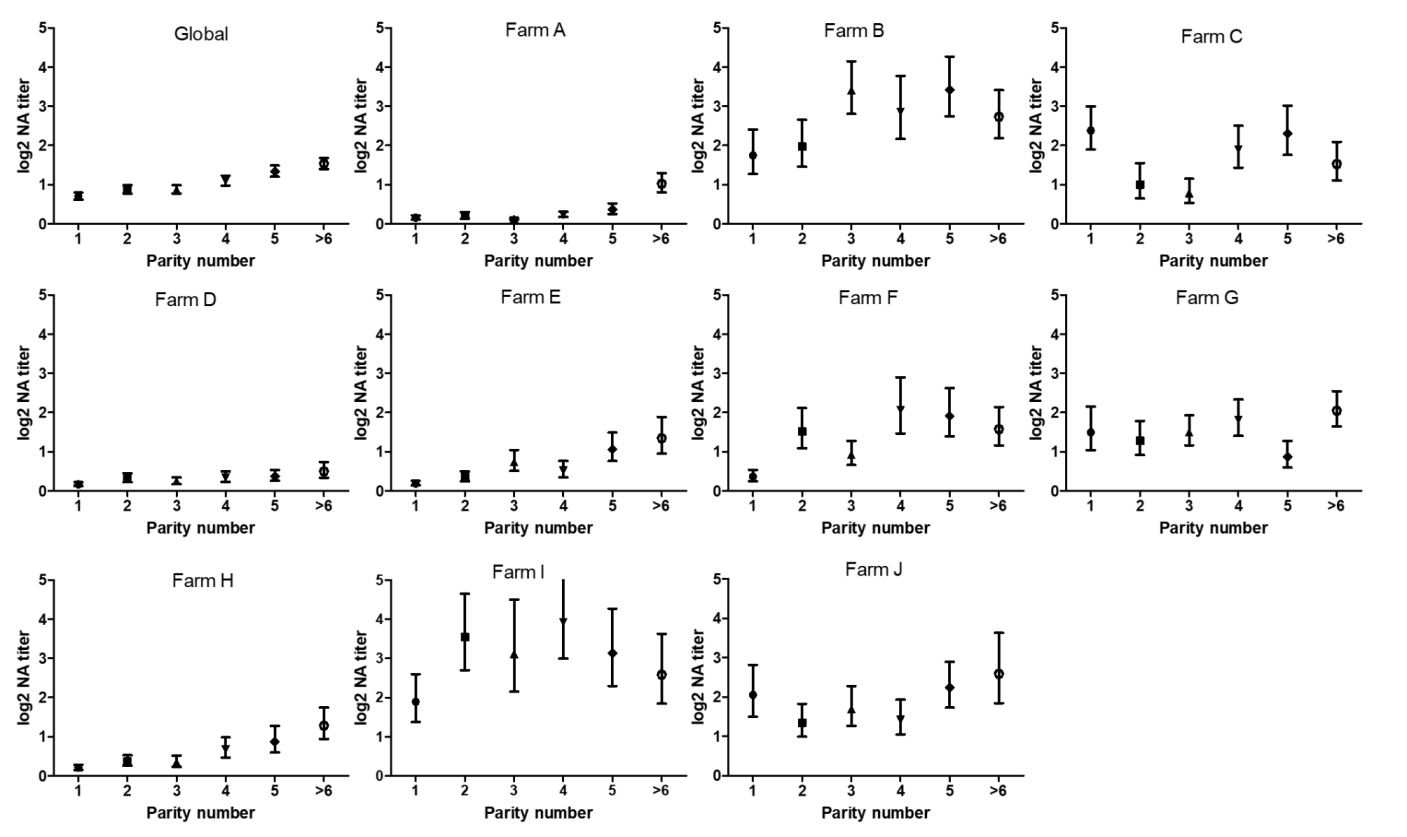
GMT of NAs of sows distributed by their parity number

The analysis by parity of the GMT of PRRSV NAs of all sows, regardless their farm of origin, revealed that, in general, the titer of NAs increases steadily, although moderately, with parity (Fig. 6.). Thus, the GMT of NAs of the young sows (i.e. parities 1–3), considered together and regardless the farm, were below the detection limit of the VN assay (0.70; 0.87 and 0.87, respectively), while the values recorded for mature sows (parity ≥ 4) were slightly higher (1.10, 1.34 and 1.53, respectively). These differences were statistically significant (*p* < 0.05).

However, when the study was carried out by farm, important differences were detected in the evolution of the titers of NAs against PRRSV by parity and no common patterns could be identified (Fig. 6). Nonetheless, when the epidemiological situation of the farms was included in the analysis, PRRSV stable and no-outbreak farms maintained a common pattern, with generally low GMT of NAs against PRRSV and a steady increase in PRRSV-NA titers with the sow parity. On the contrary, those farms that had experienced a recent outbreak and/or were considered unstable to PRRSV, exhibited a great variability in the distribution of the GMT of NAs across parities.

## Discussion

Highly variable viruses are usually difficult to control. Although different factors might contribute to this difficulty, the lack of recognition of heterologous viruses by the previously existing immunity hinders the development of universal vaccines, able to confer protection against the myriad of possible viral variants. In the last decades, the discovery of individuals that develop NAs capable of recognizing a variety of antigenically distant isolates of viruses as different as HIV [17], IV [18] or HCV [20] has aroused a great interest due to their potential for the development of more efficacious control strategies against those pathogens. Consequently, their study, initially limited to HIV, has been expanded to other viral pathogens as the already mentioned IV and HCV or the recently emerged SARS-CoV-2 [32].

In the case of PRRSV, it is well known that its genome exhibits one of the highest variabilities among RNA viruses [7] and elicits a poor and slow-developing immune response. Both characteristics, together, lead to a poor recognition of heterologous isolates by already infected or immunized pigs [15]. However, some studies carried out in the last decade have confirmed the appearance of elite neutralizers upon PRRSV infection as supported by the broad reactivity of their PRRSV-specific NAs. However, it is noteworthy that the definition of bNA is unclear in the case of PRRSV and the different studies have followed different approaches in relation to the number and type of isolates used in the VN assays, the type of VN assay performed and the designated titer of NAs set as the cutoff. Thus, in the case of PRRSV-2 a field study carried out using sera obtained from sows exposed to multiple PRRSV strains and an ELISA-based VN assay pointed to the existence of a high proportion of individuals with bNAs [16]. On the contrary, an experimental study carried out in growing pigs either infected with a field strain or vaccinated and challenge with the same field strain indicated that only 1 out of 176 pigs was able to recognize all viruses used in the study [26], although it should be mentioned that one of the viruses included in the panel was a PRRSV-1 isolate and the cut-off value to define bNAs was very stringent (i.e. 4 log_2_).

In the case of PRRSV-1 an experimental study carried out using hyperimmune monospecific sera showed that up to 10% of the sera contained bNAs, defined as sera that recognized all 39 viruses used in that panel and had a GMT of NAs ≥ 4 log_2_ [15]. However, more recently, a field study has suggested that bNAs against PRRSV-1 are rare in the sow population of endemically infected farms [33].

Altogether, the abovementioned studies indicate a significant variability in the proportion of elite neutralizers in the case of PRRSV. However, it should be kept in mind that the experimental studies might not represent the actual patterns of PRRSV exposure under field conditions and that the scarce field studies available are limited to a low number of farms, which can condition the representability of the results. Thus, previous studies carried out with other viruses indicate that different factors, such as the variety of strains to which the individuals have been exposed [34] and the genetic background of the individuals [35], among other factors, might play a role in the elicitation of bNAs.

Thus, the objective of the present study was to deepen the knowledge of PRRSV-1 elite neutralizers. Specifically, we pursued to determine the frequency of sows exhibiting bNAs against PRRSV-1 under field conditions and to characterize the epidemiological factors that could have an influence on the frequency of elite neutralizers. To fulfill these objectives, a total of 11 PRRSV-1 positive farms were selected and the genetic background of the sows, the vaccination protocol implemented in each farm, the PRRSV stability status and their PRRS outbreak history were taken into account in order to decipher whether any of these parameters has an influence on the cross-reactivity of PRRSV-specific NAs.

For the purpose of this study, an elite neutralizer was defined as an individual whose NAs were able to recognize all PRRSV-1 viruses included in the VN assays and its GMT of NAs was ≥4 log_2_. This cut-off value was selected on the basis of the results of previous studies that determined that the passive transfer of NAs sufficient to achieve a titer of 1:16 (i.e. 4 log_2_) in sera was sufficient to confer sterilizing immunity in sows challenged in the last third of gestation [36]. However, a more stringent criterion was also applied for the study of the global proportion of elite neutralizers in the studied population and an additional cut-off value of GMT of NAs of 6 log_2_ was also analyzed. This value corresponds to the titer of NAs necessary to prevent viremia after passive transfer in naïve piglets [37]. Nonetheless, as the present study has focused on the sow population, most analyses have been carried out using a GMT of NAs = 4 log_2_ as the cut-off to determine elite neutralizers.

When all sows sampled, regardless of their farm of origin, were considered in the analysis, the GMT of NAs against all the viruses used in the VN panel, including one PRRSV-2 isolate, was 2.12 log_2_. This value was slightly higher when the PRRSV-2 isolate was excluded from the panel (i.e. 2.43 log_2_). These values can be considered low, as they are well below the titer necessary to provide protection against the reproductive failure [36]. Although the actual GMT of NAs in the population against a particular PRRSV isolate might vary, these low mean values confirm that the recognition of heterologous strains by PRRSV-specific NAs elicited by previous exposures to the virus tends to be low. This poor recognition of heterologous viruses by PRRSV NAs might contribute to explain the recurrent PRRS outbreaks caused by lateral PRRSV introductions that are so commonly reported in PRRSV-positive farms [38], although other factors, such as a low level of cell-mediated immunity, which has been related to protection [39], and the virulence of the secondary PRRSV isolate, which might play a role in evading the previously existing immunity [22], might also play a role and contribute to explain the repeated outbreaks observed in the field.

The low GMT of NAs found in this study is consistent with the low proportion of sows classified as elite neutralizers. Thus, roughly 7.0% of the sows neutralized all viruses of the panel and had a GMT of NAs equal or higher than 4 log_2_ and only five sows (i.e. 0.76%) reached a GMT of NAs of 6 log_2_. However, when the PRRSV-2 isolate was excluded from the panel the proportion of elite neutralizers increased to approximately 15% when the GMT of NAs cut-off value was set at 4 log_2_ and up to 18 sows (i.e. 2.74%) reached a GMT of NAs of 6 log_2_. These proportions are similar to those found under experimental conditions when only PRRSV-1 isolates were included in the viral panel [15]. On the other hand, the lower proportion of elite neutralizer when a single PRRSV-2 isolate was included in the analyses confirms that PRRSV-1 and PRRSV-2 are very different not only at the genomic [6] but also at the antigenic level, as it has been previously reported [12, 40] and could help to explain the lack of protection between both PRRSV species observed in experimental studies [41, 42]. Finally, when the more stringent conditions used by Trible et al., 2005 [25] were applied in our analysis (i.e. all isolates of the panel should be neutralized at titers ≥4 log_2_) the proportion of elite neutralizers decreased to 0.6%, a value very similar to that found by those authors under experimental conditions.

When the data were analyzed by farm, significant differences were found in the GMT of NAs and in the proportion of elite neutralizers depending on the farm characteristics. Thus, it was observed that PRRSV unstable farms and farms that had suffered a PRRS outbreak in the last 12 months had a higher GMT of NAs than stable farms and farms without outbreaks (i.e. 0.73 vs. 1.84 in stable and unstable farms, respectively). These results seem to confirm the theory that repeated exposures to the virus play an important role in the generation of bNAs and are consistent with those of previous studies carried out under field conditions by other authors in farms infected with PRRSV-1 or PRRSV-2. Thus, in the case of PRRSV-2, Robinson et al. (2015) [16] reported exceptionally high NA-titers (between 4 log_2_ and 8 log_2_) against nine heterologous isolates in sows of farms that had experienced severe and recurrent outbreaks and/or were repeatedly and intentionally exposed to PRRSV to protect them. On the contrary, and in the case of PRRSV-1, Martín-Valls et al. (2023) [33] have proposed that PRRSV-specific bNAs are less likely to develop in endemically infected farms, where sows are only exposed to vaccine viruses or to the resident PRRSV strain and limited introductions of other isolates are observed.

The relevance of repeated antigenic exposures for the development of bNAs has been described for other viruses as HIV. Thus, Cortez et al. (2012) [34] determined studying HIV superinfected and single infected women that the NAs response of superinfected women were significantly broader and more potent than that of single infected women and that the proportion of elite neutralizers increased from 1 to 17%. In our study the proportion of elite neutralizers increased from 1.34% in stable farms to 11.67% in unstable farms, suggesting that continuous antigenic stimulation might be important for augmenting the heterologous recognition capability of the NAs elicited also in the case of PRRSV. Even more, the approach of exposing pigs sequentially to PRRSV of different species and subtypes has been recently followed successfully for the induction of bNAs that recognized PRRSV-1 and PRRSV-2 isolates [43]. Although the mechanism that explains the broader recognition of isolates achieved upon repeated exposures, preferentially to different viruses, has not been elucidated, it has been proposed that frequent exposures to heterologous viruses could specifically enhance the response to conserved and probably poor immunogenic epitopes and, in the case of PRRSV-1, surpass the potent response against neutralizing epitope of GP4, which is considered a decoy epitope [14, 44].

Furthermore, the quality of the NA response in a particular farm will also be dependent on the characteristics of the isolates that produce each of the registered outbreaks or infections. Unfortunately, the current study lacks information pertaining to which PRRSV strains were circulating in the selected farms. The data about historical viral circulation was derived from an epidemiological survey, and neither the circulating strains nor their sequences were available. The absence of this crucial information hinders the ability to discern whether bNA response is linked solely to exposure to multiple strains or is influenced by specific strains. In this line of thinking, previous studies have highlighted the existence of isolates with distinctive characteristics that can condition the quality of the host’s humoral response, such as isolates with a heightened capacity to induce bNAs [21, 45]. Even more, it has been demonstrated that exposure to highly virulent PRRSV, either through natural infection or serum inoculation, can affect cross-neutralization capacity of the generated NAs [16]. We cannot provide information on the type of viruses circulating at the time of sampling, nor on their phylogenetic relationships or with the viruses included in the panel. This information could have helped interpret the results obtained more precisely. However, it should be acknowledged that nucleotide sequence alone may not accurately predict the extent of cross-neutralization as the molecular bases on PRRSV NA-cross-reactivity are yet to be deciphered [46]. Therefore, data additional to the sequences of circulating strains are needed, e.g. in vivo studies, to establish a robust cause-effect relationship between the characteristics of the circulating isolates and their cross-neutralization capability. In this line of thinking, the antigenic similarity between the combination of isolates that infect a single animal may play a role in the secondary response elicited (unpublished results) and in the breath of the response.

Another potential weakness of our study is the possible misclassification of some farms. In our study, farms were classified as stable when no PRRSV were detected in any of the 30 blood samples taken from each due to wean pig batch during the previous 90 days. However, in the face of a low PRRSV prevalence in breeding sows, it is possible that some positive pig batches might have gone unnoticed and that some unstable low-prevalence farms could have been misclassified as stable. Nonetheless, the viral circulation in these farms is considered to be generally low and unlikely to elicit a strong immune response [33]. Thus, the general rule that repeated exposures might enhance cross-reactivity still applies.

On the other hand, if repeated exposures to the virus are a key factor for the development of bNA, the NA response is expected to increase with the age. In our study, we observed that, indeed, the GMT of NAs gradually increased, although moderately, with the parity of the sows. This fact has also been observed in other viral infections where bNA response is developed only after several years of infection and is associated with the accumulation of somatic mutations of the antibodies against conserved neutralizing epitopes [47]. However, parity number has not been observed to play significant role in the development of PRRSV bNAs in other studies [33] and its importance should be further studied. In fact, the effect of the parity number on bNA response was observed quite systematically in stable farms but not necessarily in unstable farms or farms with recent outbreaks. This may be due to the fact that, under stable conditions, the bNA titers would rise due to the above described mechanism of somatic mutations of NAs against conserved epitopes whereas in unstable farms the exposure of the whole population to new viral isolates would lead to higher mean titers due to a secondary response in all re-infected animals, regardless of their parity number.

Besides the age, genetics could have an effect in the development of PRRSV-specific NAs. In fact, between-breed differences in anti N protein antibody response to PRRSV infection have been identified [48, 49]. However, there is not much information in relation to the NA response associated to certain purebred or crossbreeds. In this study no significant differences could be detected between different genetic lines, although the sample size might have not been sufficient to ensure this lack of correlation. Nonetheless, it is remarkable that some particular individuals, regardless of all other studied factors, exhibited an outstanding heterologous recognition and neutralization capability. Although other factors might have an influence on the final outcome, the individual NA response is conditioned by the genetic characteristics of the host, which determine the sequence of epitopes that they are able to recognize. This theory would explain why the breadth of the NA response is not a general fact and has an individual component. This fact would imply that the combination of several viral infections and animal haplotype is necessary to obtain an optimal NA response [43]. However, the characterization of the sow haplotype was beyond the scope of this study and further investigations should be conducted to clarify this point.

Finally, as the use of PRRSV vaccines to control the disease is a common practice in the field, two vaccine strains that are frequently used in control programs were included in the panel of viruses analyzed. The NA titer obtained against field isolates in farms that used Vac-1 was higher than that obtained in farms that used Vac-3, although the differences were not statistically significant. This higher titer might result from incorporating in the panel isolates more closely related to the original Vac-1 vaccine strain than to Vac-3. However, it’s worth noting that there is also an isolate closely related to the original Vac-3 vaccine strain. Additionally, as already mentioned, the nucleotide sequence may not accurately predict the extent of cross-neutralization or the conferred protective immunity [50]. On the other hand, it is noteworthy that vaccinated animals had higher NA titers against both vaccine viruses than against all other viruses, a phenomenon that has been previously reported [33]. Notably, NAs against the vaccine strain were not detectable in 10% and 20% of the sows vaccinated with Vac-1 and Vac-3, respectively. A similar percentage of animals without detectable levels of NAs against vaccine virus has been reported in vaccinated farms by Martín-Valls et al. (2023) [33] confirming the unresponsiveness of a small proportion of animals. This phenomenon has previously been described using different serological tests in the case of PRRSV vaccinated or infected sows and is considered a common feature in PRRSV infection [51, 52]. However, the ultimate reasons for this lack of responsiveness remain unclear.

## Conclusion

The results of this study indicate that the titers of NAs against heterologous isolates elicited by PRRSV infection under field conditions are relatively low and, in most cases, below the titers considered necessary to confer protection against reproductive failure. However, a low proportion of sows, 15% on average, can be considered elite neutralizers and are able to recognize efficiently a variety of PRRSV. The repeated exposures to PRRSV seem to play a very important role in the elicitation of those bNAs exhibited by the elite neutralizers, as they are much more frequent in unstable farms and farms that have experienced recent outbreaks. In contrast, parity only marginally augments the titer of bNAs in stable farms, indicating a limited role in the breadth of PRRSV-specific neutralizing antibodies, especially when compared to the impact of PRRSV-exposure history.

### Electronic supplementary material

Below is the link to the electronic supplementary material.

Supplementary Material 1

Supplementary Material 2

## Data Availability

The datasets used and/or analyzed during the current study are available from the corresponding author on reasonable request.

## Abbreviations

PRRS: Porcine reproductive and respiratory syndrome
PRRSV: Porcine reproductive and respiratory syndrome virus
bNAs: Broadly neutralizing antibodies
VN: Virus neutralization
GMT: Geometric mean of the titer
MLV: Modified live viruses
FBS: Fetal bovine serum
