# Supplementary figures and images for: Determination of the frequency of individuals with broadly cross-reactive neutralizing antibodies against PRRSV in the sow population under field conditions
Source: Porcine Health Manag. 2024 Jul 8;10:26. doi: 10.1186/s40813-024-00372-y (PMC11229297; doi:10.1186/s40813-024-00372-y)

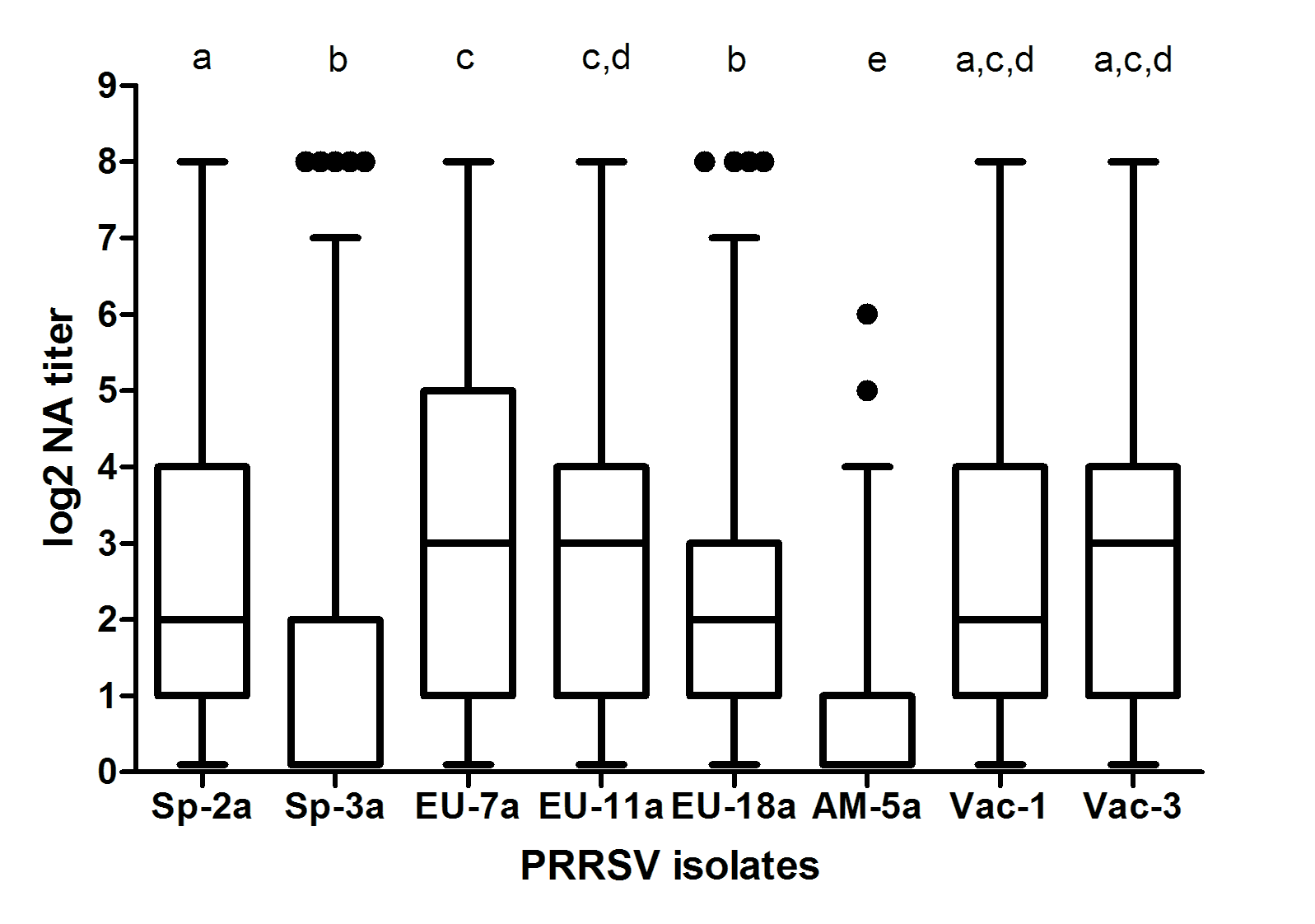

Supplement: Supplementary file 1 — Supplementary Material 1 [file 40813_2024_372_MOESM1_ESM.tif]
