# Supplementary material for: Determination of the frequency of individuals with broadly cross-reactive neutralizing antibodies against PRRSV in the sow population under field conditions
Source: Porcine Health Manag. 2024 Jul 8;10:26. doi: 10.1186/s40813-024-00372-y (PMC11229297; doi:10.1186/s40813-024-00372-y)
Supplement: Supplementary file 2 — Supplementary Material 2 [file 40813_2024_372_MOESM2_ESM.docx]

**Additional file 1. Pairwise comparison of the nucleotide sequences of ORF5 of the PRRSV isolates**

| PRRSV  isolates | Sp-2a | Sp-3a | EU-7a | EU | EU-18a | AM-5a | Vac-1 | Vac-3 |
| --- | --- | --- | --- | --- | --- | --- | --- | --- |
| Sp-2a | ID | 97.8 | 89.6 | 93.2 | 87.1 | 60.8 | 99.6 | 93.7 |
| Sp-3a |  | ID | 88.7 | 92.2 | 85.9 | 60.8 | 97.5 | 92.4 |
| EU-7a |  |  | ID | 89.1 | 85.4 | 62.2 | 89.6 | 88.2 |
| EU-11a |  |  |  | ID | 86.9 | 61.6 | 93.2 | 97.5 |
| EU-18a |  |  |  |  | ID | 61.6 | 86.7 | 86.3 |
| AM-5a |  |  |  |  |  | ID | 60.6 | 61.9 |
| Vac-1 |  |  |  |  |  |  | ID | 93.3 |
| Vac-3 |  |  |  |  |  |  |  | ID |
